# Association of pre-ESRD care education with patient outcomes in a 10-year longitudinal study of patients with CKD stages 3–5 in Taiwan

**DOI:** 10.1038/s41598-021-01860-9

**Published:** 2021-11-19

**Authors:** Chu-Lin Chou, Chi-Hsiang Chung, Hui-Wen Chiu, Chia-Te Liao, Chia-Chao Wu, Yung-Ho Hsu, Wu-Chien Chien

**Affiliations:** 1 Division of Nephrology, Department of Internal Medicine, Tri-Service General Hospital, National Defense Medical Center, Taipei, Taiwan; 2 Division of Nephrology, Department of Internal Medicine, School of Medicine, College of Medicine, Taipei Medical University, No. 250, Wuxing Street, Xinyi District, Taipei City, 110 Taiwan; 3 Taipei Medical University-Research Center of Urology and Kidney, Taipei Medical University, Taipei, Taiwan; 4 Division of Nephrology, Department of Internal Medicine, Shuang Ho Hospital, Taipei Medical University, New Taipei City, Taiwan; 5 Division of Nephrology, Department of Internal Medicine, Hsin Kuo Min Hospital, Taipei Medical University, Taoyuan City, Taiwan; 6 School of Public Health, National Defense Medical Center, Taipei, Taiwan; 7 Taiwanese Injury Prevention and Safety Promotion Association, Taipei, Taiwan; 8 Graduate Institute of Clinical Medicine, College of Medicine, Taipei Medical University, Taipei, Taiwan; 9 Department of Medical Research, Shuang Ho Hospital, Taipei Medical University, New Taipei City, Taiwan; 10 Department of Medical Research, Tri-Service General Hospital, National Defense Medical Center, 7115R, No. 325, Section 2, Cheng-Kung Road, Neihu District, Taipei City, 11490 Taiwan, Republic of China; 11 Graduate Institute of Life Sciences, National Defense Medical Center, Taipei, Taiwan

**Keywords:** Kidney diseases, Patient education, Outcomes research, Chronic kidney disease

## Abstract

There is little comprehensive education for people with end-stage renal disease (ESRD) progress. We investigated the differences in terms of outcomes between patients with CKD stages 3–5 who enrolled and did not enroll in the pre-ESRD care education in Taiwan. This retrospective cohort study was conducted using data from the National Health Insurance Research Database (NHIRD). All patients diagnosed with CKD stages 3–5 who received the pre-ESRD care education through the pay for performance (P4P) program were enrolled. Based on whether or not they participated in the program, they were categorized into P4P or non-P4P groups. All analyses were performed from January 2006 through December 2015. Study outcomes were risk of hemodialysis dependency, hospitalization, and all-cause mortality. In this study of 29,337 patients, those with CKD stages 3–5 in the P4P group had lower events of hemodialysis, hospitalization, and all-cause mortality compared to patients in the non-P4P group. This study suggested that pre-ESRD care education is associated with increased patient outcomes, resulting in lower hemodialysis and hospitalization events and a higher overall survival rate in patients with CKD stages 3–5. Patient education could raise opportunities to improve pre-ESRD care by reaching patients outside the traditional health care setting.

## Introduction

The prevalence of chronic kidney disease (CKD) in Taiwan is approximately 11.9% higher and affects more than 2.5 million people^1^. Recent data on prevalence rates of CKD stages in Taiwan showed that CKD 3A, 3B, 4, and 5 were 8.3%, 1.9%, 0.3%, and 0.2%, respectively^2^. A systematic review and meta-analysis of 100 observational studies indicated that global prevalence of CKD stage 3, CKD stage 4, and CKD stage 5 were 7.6% (6.4–8.9%), 0.4% (0.3–0.5%), and 0.1% (0.1–0.1%), respectively^3^. CKD has a high global prevalence with a constant estimated global prevalence of CKD with majority stage 3^3^. Therefore, CKD is a global health issue with a high economic cost associated with increased cardiovascular morbidity, early mortality, and decreased life quality^4^.

It has been widely reported that CKD with end-stage renal disease (ESRD) progress is primarily associated with accelerated hypertension, diabetes mellitus (DM), gout, primary renal disorders, older age, and drug side effects^5^. Moreover, CKD progression with reduced glomerular filtration rate has been associated with increased cardiovascular disease (CVD) risk^4,6^, greater severity of vascular disease^7–9^, increased all-cause, and CVD mortality^10,11^, acute stroke^12,13^, and increased mortality in heart failure^14–16^. It is crucial to ameliorate a decrease in the glomerular filtration rate and educate CKD patients to prevent kidney disease progress through multidisciplinary care education programs.

In terms of CKD prevention and treatment, the main goal is to slow the CKD progression to ESRD and its subsequent adverse effects through early CKD diagnosis and control of the underlying causes. In addition to these strategies, multidisciplinary care education programs have been widely adopted during CKD treatment, and they resulted in a more effective therapy, slowing the CKD progression to ESRD, and improving dialysis quality^17–20^. Thus, it is crucial to include care education programs in the prevention and treatment of CKD.

Pay for performance (P4P) programs used for pre-end-stage renal disease (pre-ESRD) care education in Taiwan is a proper strategy and a promising approach through value-based purchasing on incentives and renal indicators to improve health care quality and disease prognosis for CKD stages 3–5 patients since 2006^21,22^. In other words, the P4P is an encouraging approach to improving health care quality and self-awareness education by connecting financial incentives to supplier performance. The rationale for the initiative is that by explicitly paying for recommended care, quality improvement can be promoted, resulting in better patient outcomes^21,22^. Under this financial incentive program, nephrologists are asked to provide care by more closely following clinical guidelines and by a multidisciplinary care team^21,22^. Before P4P programs began, health care providers were traditionally involved in improving CKD care through multidisciplinary care education programs without financial incentives^23^. This multidisciplinary care has become a focal point for discussing the cases of different professionals with the ultimate goal of establishing a consensus on the diagnosis, education, evaluation, and treatment of patients^24–28^. In addition, as reported previously, multidisciplinary care education programs have been introduced to assist quality of care, including CKD, dialysis, chronic obstructive pulmonary disease, and coronary artery disease, and DM in many countries^17,29–34^. However, there are few studies on the efficiency of pre-ESRD care education with P4P programs in improving the pre-dialysis outcomes. In this study, using the National Health Insurance Research Database (NHIRD), we explored the outcomes of pre-ESRD care education in patients with CKD stage 3–5 who benefited of the P4P program in Taiwan.

## Methods

### Data sources

The ethical committee, the Institutional Review Board of the Tri-Service General Hospital, approved this retrospective study (TSGH IRB No. B-109-10) and all experimental protocols. Also, informed consent was waived by the ethical committee of the Tri-Service General Hospital. All methods were carried out in accordance with relevant guidelines and regulations. The Taiwan National Health Insurance system is a universal single-payer insurance system and enrolled all these insurance schemes into a single national insurance system. The NHIRD provides longitudinal databases with a detailed description of the database guidelines and regulations which randomly sampled two million beneficiaries from the original NHIRD^35^. The representativeness of NHIRD has been validated by Taiwan’s National Health Research Institutes^35^. In this study, we obtained the original data of Taiwan NHIRD, which ranged between 2000 and 2015, to investigate the outcome of the P4P programs for pre-ESRD care education in patients with CKD stage 3–5 since 2006. This study enrolled all patients diagnosed with CKD stages 3–5 who received the P4P program linked with the NHIRD. All the target patients were identified based on the Clinical Modification (ICD-9-CM) codes determined by the International Classification of Diseases (Ninth Revision). If patients with CKD stages 3–5 had enrolled in the P4P program before dialysis, they were allocated to the P4P group. The information related to the cohort identification was collected for a period of 10 years, specifically from 2006 to 2015.

### Study design and outcomes

Using the Taiwan NHIRD, the subjects were enrolled in the following two groups: P4P and non-P4P. This study was conducted using the data from the inpatient and outpatient claims recorded between 2006 and 2015. The P4P group comprised patients with CKD stages 3–5 who received the complete P4P program and had no dialysis records. The control group, referred to as the non-P4P group, included patients with CKD stages 3–5 who did not receive the complete P4P program and had no dialysis records in the database.

We excluded patients whose age and sex was not recorded, who were aged < 40 years, for whom a minimum of two years of data was unavailable after the initiation of the program, who had a history of cancer and human immunodeficiency virus, and who received renal replacement therapy and kidney transplantation before the initiation of the P4P program. The last exclusion criterion was applied to ensure that ESRD with renal replacement therapy occurred only after the initiation of the P4P program.

The index date was defined as the onset date of the P4P program initiation. The study follow-up period started from this date and lasted until the onset date of ESRD, with the endpoints being the occurrence of dialysis therapy or kidney transplantation, hospitalization, and mortality events due to any cause. We assigned a date to the control patients who did not receive the P4P program, which matched their corresponding case patients (referred to as the index date).

Finally, in this cohort study, cases and controls were matched according to age, gender, socioeconomic status, CKD stages 3–5, comorbidities (DM, hypertension, hyperlipidemia, myocardial infarction, congestive heart failure, cerebrovascular disease, chronic pulmonary disease, chronic liver disease, peptic ulcer, and dementia), and medications used in the six months before program initiation (specifically: metformin, sulfonylureas, thiazolidinediones, α-glucosidase inhibitors, dipeptidyl peptidase 4, insulins, angiotensin-converting enzyme inhibitors/angiotensin receptor blockers, beta-blockers, calcium channel blockers, diuretics, antiplatelet drugs, statins, nonsteroidal anti-inflammatory drugs, and steroids). The incidence density sampling approach was used to match controls with each case according to age (± 1 year), sex, and the follow-up period of the P4P program initiation. This approach allowed the observation of both patient groups during similar periods, thereby eliminating the bias caused by differences in sampling time.

The study outcomes were the rate of dialysis dependency, hospitalization, and mortality. Dialysis dependency was measured from the date of dialysis initiation for at least three consecutive months or from the approval date appearing on the catastrophic illness certificate for ESRD, whichever occurred first, to ensure the actual need of dialysis and its administration. The dialysis modes included hemodialysis (HD) (ICD-9-CM procedure codes: 58027C, 58029C), and the participants were followed up to their first hospitalization. The outcome of interest was the first hospitalization for any cause reported in the NHI claim database after study initiation. Mortality within the 10-year follow-up period was considered noteworthy. Also, the causes of mortality after study initiation—such as ischemic heart disease, heart failure, arrhythmia, out-of-hospital cardiac arrest, cerebrovascular accident, infectious disease, and DM complications—were assessed based on the primary diagnostic codes entered in the inpatient and emergency claims 30 days before death. Patients were followed up from the index date to the earliest of the following events: outcome occurrence, death, disenrollment from the NHI program, or study end date (December 31, 2015).

### Review of the P4P program for pre-ESRD care education

To enhance the quality of CKD care, the NHI Administration in Taiwan initiated the P4P program for pre-ESRD care education in 2006. The program’s multidisciplinary care members include nephrologists, nurses and dietitians and it enrolled patients with indications including individuals with CKD stage 3b (glomerular filtration rate (GFR) ~ 30–44.9 mL/min/1.73 m^2^) or CKD stage 4 (GFR ~ 15–29.9 mL/min/1.73 m^2^) or CKD stage 5 (GFR < 15 mL/min/1.73 m^2^), and heavy proteinuria regardless of CKD stage (24-h urine total protein excretion > 1,000 mg or urine protein and creatinine ratio > 1,000 mg/g). Besides offering an appropriate CKD education knowledge, the multidisciplinary care team could track the patient health status and provide nephrologists and family members with the necessary information and communication. The care indicators included renal function maintenance, improvement of proteinuria, continuous multidisciplinary care, pre-inserted HD access, and CKD management and education.

### Covariates

Baseline demographics and clinical characteristics were recorded before obtaining the index date. Socioeconomic status was based on monthly income calculated from the insurance premium provided in the patient enrollment profile, and it was divided into six categories. CKD stages were divided into 3, 4, and 5 based on the ICD code. The comorbidities included DM, hypertension, hyperlipidemia, myocardial infarction, congestive heart failure, cerebrovascular disease, chronic pulmonary disease, chronic liver disease, peptic ulcer, and dementia. We collected the data of patients who had received medications within six months before the index date. The prescribed medications included DM and non-DM medications; among DM medications were insulins, biguanides, sulfonylureas, meglitinides, α-glucosidase inhibitors, and thiazolidinediones; while the non-DM medications included angiotensin-converting enzyme inhibitors/angiotensin receptor blockers, nonselective and selective beta-blockers, calcium channel blockers, loop diuretics, aspirin, and statins. The ICD-9-CM disease diagnostic codes used for previous or coexisting diseases, and the Anatomical Therapeutic Chemical codes used for medications are listed in Supplemental Table 1.

### Statistical analysis

Statistical analyses were performed in S.A.S. (S.A.S. System for Windows, version 9.1.3; S.A.S. Institute, Cary, NC, USA). The χ^2^ test and *t*-test were used to analyze and evaluate the differences in age or comorbidities and medications between the P4P and non-P4P groups to assure proper matching. Fisher's exact test was applied for categorical variables. The Cox proportional regression hazards model was used to compare the incidence rates of dialysis dependency/kidney transplant, hospitalization, and mortality between the two groups, after modifying comorbidities. The Kaplan–Meier method and the log-rank test were used to estimate the outcomes of the two groups. A two-tailed *P* value < 0.05 was considered statistically significant.

## Results

### Study selection

**Figure 1:**
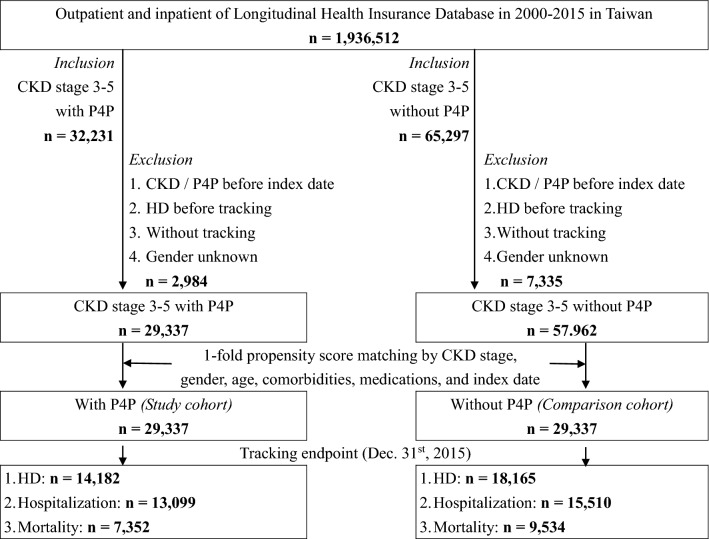
Cohort Assembly in This Study. Abbreviations: CKD = chronic kidney disease; HD = hemodialysis; P4P = pay for performance program.

This study enrolled a total of 32,231 individuals diagnosed with CKD stages 3–5 who received the P4P program for the first time, and 2,894 of these were excluded (specifically, those with CKD stages 3–5/P4P before the index date, HD before tracking, without tracking, and of unknown gender). After applying the exclusion criteria and the propensity score matching according to CKD stage, gender, age, comorbidities, medication, and index date, a total of 29,337 patients with matched controls participating in the P4P program were enrolled in this study, as shown in Fig. 1. During the 10-year follow-up study, we identified 14,182 HD events (48.3%), 13,099 hospitalization events (44.6%), and 7,352 mortality events (25.0%) in the P4P group and 18,165 HD events (61.9%), 15,510 hospitalization events (52.8%), and 9,534 mortality events (32.5%) in the control group.

### Study characteristics

**Figure 2:**
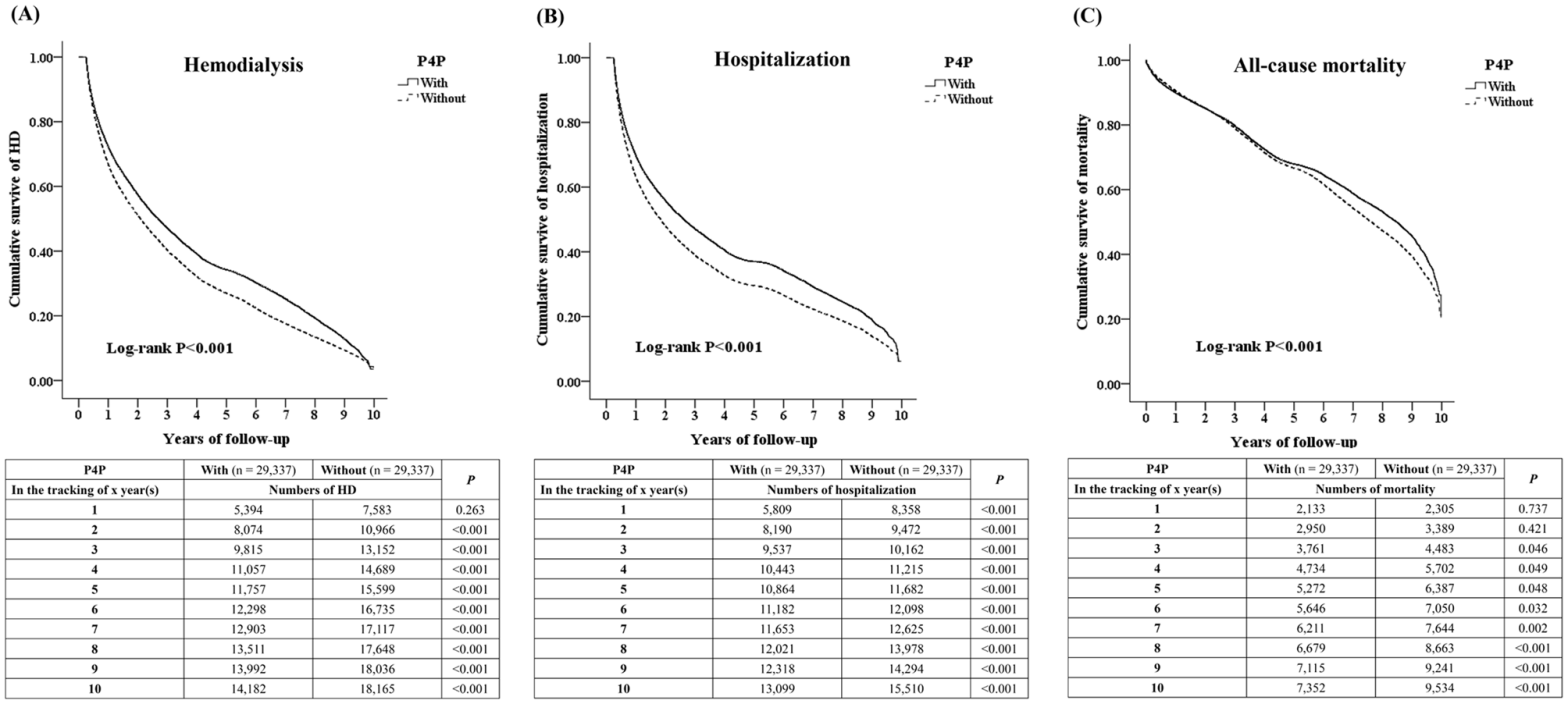
Kaplan–Meier Curves for the Cumulative Incidence of (**A**) Hemodialysis, (**B**) Hospitalization, and (**C**) All-cause Mortality with Log-Rank Test in Patients with CKD Stages 3–5 Enrolled or Not in the Pay for Performance Program Intervention.

The baseline characteristics of P4P patients with CKD stages 3–5 and those of controls are shown in Table 1. No differences were observed in gender, age, insurance premiums, comorbidity, and medication between the groups. The P4P group was associated with a significantly decreased risk of HD, hospitalization, and all-cause mortality events (log-rank *P* < 0.001), as shown by the respective Kaplan–Meier curves for cumulative risks in Fig. 2. The HD events were lower in the P4P patients than in the control group from the second year of follow-up to the 10th year.

**Table 1:** Baseline characteristics of study subjects.

P4P	Total		With		Without		*P*
Variables	n	%	n	%	n	%	
Total	58,674	100	29,337	50	29,337	50	
**CKD stage**							0.999
3	35,504	60.51	17,752	60.51	17,752	60.51	
4	16,372	27.90	8,186	27.90	8,186	27.90	
5	6,798	11.59	3,399	11.59	3,399	11.59	
**Gender**							0.999
Male	33,728	57.48	16,864	57.48	16,864	57.48	
Female	24,946	42.52	12,473	42.52	12,473	42.52	
Age (years)	68.01 ± 13.43		67.95 ± 13.01		68.06 ± 13.84		0.321
DM	14,149	24.11	7,053	24.04	7,096	24.19	0.678
HT	15,104	25.74	7,503	25.58	7,601	25.91	0.355
Hyperlipidemia	1,452	2.47	730	2.49	722	2.46	0.832
Stroke	4,931	8.40	2,445	8.33	2,486	8.47	0.542
CAD	1,997	3.40	1,008	3.44	989	3.37	0.665
HF	4,989	8.50	2,503	8.53	2,486	8.47	0.801
PVD	6,517	11.11	3,220	10.98	3,297	11.24	0.312
Chronic pulmonary disease	5,182	8.83	2,585	8.81	2,597	8.85	0.861
Chronic liver disease	5,097	8.69	2,564	8.74	2,533	8.63	0.650
Dementia	955	1.63	499	1.70	456	1.55	0.161
ACEI/ARB	5,464	9.31	2,795	9.53	2,669	9.10	0.073
β2 blocker	2,209	3.76	1,098	3.74	1,111	3.79	0.778
CCB	5,933	10.11	2,978	10.15	2,955	10.07	0.753
Antiplatelet drug	1,782	3.04	888	3.03	894	3.05	0.885
Statin	1,415	2.41	704	2.40	711	2.42	0.851
NSAID	8,990	15.32	4,562	15.55	4,428	15.09	0.125
Steroid	17,493	29.81	8,711	29.69	8,782	29.93	0.522
DPP4is	5,639	9.61	2,828	9.64	2,811	9.58	0.812
Metformin	3,964	6.76	1,986	6.77	1,978	6.74	0.895
TZD	6,847	11.67	3,411	11.63	3,436	11.71	0.748
Sulfonylureas	5,986	10.20	2,975	10.14	3,011	10.26	0.623
α-Glucosidase inhibitors	6,118	10.43	3,034	10.34	3,084	10.51	0.499
Insulin	14,149	24.11	7,053	24.04	7,096	24.19	0.678

*P < 0.05; statistical significance.

*Abbreviations* CAD = coronary artery disease; CKD = chronic kidney disease; DM = diabetes mellitus; HT = hypertension; HF = heart failure; PVD = peripheral vascular disease; P4P = pay for performance program; ACEI = angiotensin-converting enzyme inhibitors; ARB = angiotensin receptor blockers; CCB = calcium channel blocker; NSAID = nonsteroidal anti-inflammatory drugs; DPP4is = dipeptidyl peptidase-4 inhibitors; TZD = thiazolidinediones.

### Study outcome

The adjusted hazards ratios (aHR) of HD, hospitalization, and all-cause mortality events in patients with CKD stages 3–5 in the P4P group and the matched controls are presented in Table 2. Results showed that these patients had fewer events of HD [aHR, 0.766; 95% confidence interval CI 0.746–0.797; *P* < 0.001], hospitalization (aHR, 0.790; 95% CI 0.766–0.814; *P* < 0.001), and mortality (aHR, 0.794; 95% CI 0.766–0.823; *P* < 0.001) than the control subjects during the follow-up period, after adjusting for age, sex, insurance premium, comorbidities, urbanization level, patient care quality, area of residence in Taiwan. Regardless of CKD stages, comorbidities and medication, the P4P program proved effective, contributing to a decrease in HD, hospitalization, and all-cause mortality events.

**Table 2:** Hazard ratio of hemodialysis, hospitalization, and mortality in patients with CKD stages 3–5 in the pay for performance program in the cox model with competing risks.

Variables	HD			Hospitalization			Mortality		
	Adjusted HR	95% CI	P	Adjusted HR	95% CI	P	Adjusted HR	95% CI	P
**P4P**									
Without	Reference			Reference			Reference		
With	0.775	0.755–0.807	< .001*	0.800	0.775–0.824	< .001*	0.804	0.775–0.833	< .001*
**CKD stage**									
3	Reference			Reference			Reference		
4	1.888	1.118–2.124	< .001*	2.011	1.317–2.628	< .001*	2.436	1.359–3.190	< .001*
5	3.033	4.049–4.172	< .001*	2.789	1.662–3.849	< .001*	4.498	2.599–7.066	< .001*
**Gender**									
Male	0.919	0.899–0.939	< .001*	0.871	0.850–0.891	< .001*	1.203	1.165–1.240	< .001*
Female	Reference			Reference			Reference		
**Age (years)**									
< 50	Reference			Reference			Reference		
50–59	1.151	1.089–1.217	< .001*	0.919	0.874–0.967	.001*	1.019	0.938–1.109	0.831
60–69	1.279	1.133–1.347	< .001*	0.932	0.889–0.978	.012*	1.191	1.101–1.288	< .001*
70–79	1.455	1.383–1.530	< .001*	0.944	0.902–0.988	.019*	1.766	1.639–1.900	< .001*
80–89	1.380	1.311–1.452	< .001*	0.784	0.747–0.821	< .001*	1.819	1.689–1.960	< .001*
≥ 90	0.997	0.925–1.074	.674	0.450	0.413–0.492	< .001*	1.444	1.307–1.594	< .001*
**DM**									
Without	Reference			Reference			Reference		
With	0.916	0.890–0.944	< .001*	1.177	1.137–1.219	< .001*	0.854	0.820–0.890	< .001*
**HT**									
Without	Reference			Reference			Reference		
With	0.787	0.763–0.800	< .001*	0.304	0.294–0.314	< .001*	0.503	0.486–0.521	< .001*
**Hospitalization**									
Without	Reference			Reference			Reference		
With	0.437	0.393–0.487	< .001*	0.710	0.639–0.789	< .001*	0.107	0.077–0.151	< .001*
**Stroke**									
Without	Reference			Reference			Reference		
With	1.006	0.958–1.057	.702	0.982	0.929–1.038	0.265	1.242	1.167–1.322	< .001*
**CAD**									
Without	Reference			Reference			Reference		
With	1.047	0.999–1.084	.050	0.997	0.960–1.037	0.478	1.051	0.998–1.102	0.052
**HF**									
Without	Reference			Reference			Reference		
With	0.975	0.937–1.012	.124	0.896	0.857–0.936	< .001*	1.163	1.104–1.225	< .001*
**PVD**									
Without	Reference			Reference			Reference		
With	0.915	0.878–0.955	.003*	0.674	0.641–0.710	< .001*	0.999	0.947–1.056	0.624
**Chronic pulmonary disease**									
Without	Reference			Reference			Reference		
With	1.478	1.418–1.540	< .001*	1.072	1.029–1.118	.007	1.446	1.370–1.528	< .001*
Chronic liver disease									
Without	Reference			Reference			Reference		
With	1.179	1.133–1.228	< .001*	1.000	0.958–1.045	0.612	0.825	0.774–0.879	< .001*
**Dementia**									
Without	Reference			Reference			Reference		
With	0.789	0.727–0.855	< .001*	0.655	0.593–0.725	< .001*	0.714	0.636–0.801	< .001*

Adjusted HR: Adjusted variables listed in the table.

*P < 0.05; statistical significance.

*Abbreviations* CAD = coronary artery disease; CI = confidence interval; CKD = chronic kidney disease; DM = diabetes mellitus; HD = hemodialysis; HR = hazard ratio; HT = hypertension; HF = heart failure; PVD = peripheral vascular disease; P4P = pay for performance program.

The median follow-up periods in the P4P and control groups were 5.87 ± 6.46 and 6.91 ± 6.42 years, respectively (Table 3). The onset duration values for CKD stages 3–5 in P4P patients in relation to HD, hospitalization, and mortality events were 2.52 ± 2.44, 2.86 ± 2.70, and 3.47 ± 2.80 years, respectively. In contrast, the duration of the same events in the matched control group was 2.28 ± 2.22, 2.53 ± 2.46, and 3.43 ± 2.81 years, respectively. The P4P patients showed delayed HD, hospitalization, and all-cause mortality compared to patients in the matched control group during the follow-up course.

**Table 3:** Years to hemodialysis, hospitalization, and mortality in patients with CKD stages 3–5 with or without pay for performance program.

	Hemodialysis	Hospitalization	Mortality
	Mean ± SD	Mean ± SD	Mean ± SD
With P4P	2.52 ± 2.44	2.86 ± 2.70	3.47 ± 2.80
Without P4P	2.28 ± 2.22	2.53 ± 2.46	3.38 ± 2.82
Total	2.38 ± 2.33	2.67 ± 2.56	3.43 ± 2.81

P4P = pay for performance program; SD = standard deviation.

## Discussion

The purpose of pre-ESRD care education is to ameliorate patients’ conditions and counteract the progression of CKD-to-ESRD. Our study explored the outcomes of the P4P program for pre-ESRD care education in patients with CKD stages 3–5. After performing multivariate adjustments and subgroup analysis, our results indicated that the P4P program had beneficial effects on patient outcomes, such as lower HD, hospitalization, and all-cause mortality events.

Preventing CKD development and its complications is possible through early diagnosis and by treating any underlying disease to slow its progression. Pre-ESRD care education is an effective and beneficial strategy for CKD prognosis. Devins GM et al. found that it delayed dialysis time in a prospective, randomized and controlled trial^36^, which was similarly found in our study. The authors suggested that pre-ESRS care education is an essential approach to delay CKD progression and extend time to dialysis therapy in patients with advanced CKD^36^. A randomized trial of a pre-ESRD care education two-phase intervention (phase 1 using educational booklets and video; phase 2 using an interactive educational group session) significantly increased the self-care dialysis ability^37^. Furthermore, innovative education methods, including family and community engagement, self-management care, shared decision-making, and digital media, were also proved as additional successful tools^38^. The results of our study demonstrated again that pre-ESRD care education is essential for CKD prevention and treatment.

In relation to hospitalization risk in advanced CKD cases, a study of 170,897 patients who initiated dialysis with linked Medicare claims from the United States Renal Data System, showed a higher one-year mortality associated with pre-dialysis cardiovascular-related or infection-related hospitalization events^39^. In our study, data indicated that pre-ESRD care education effectively lowered hospitalization risk (aHR, 0.790; 95% CI 0.766–0.814; *P* < 0.001) in patients with advanced CKD. We observed that the decreased risk could be achieved through health education on dietary control, care continuity, and compliance with clinical guidelines, leading to an improved life quality and subsequent delay of complications in patients with advanced CKD.

Education is key to reducing mortality in patients with CKD. One study on patients sourced from the United States Renal Data System database showed that educational programs improved dialysis preparation and patient survival (aHR, 0.80; 95% CI, 0.68–0.94) in CKD patients^40^. Another study of 1,256,640 patients initiated on chronic dialysis sourced from the same database, also demonstrated that patients receiving pre-ESRD care education had a better survival rate than those without^41^, showing that the education program was associated with significantly higher pre-ESRD nephrologist care rates, preemptive transplant wait-listing, transplantation occurrences, and a lower mortality risk^40,41^. A meta-analysis of early versus late referrals to nephrologists indicated a significantly higher overall mortality, longer hospital stay, and early at the time of renal replacement therapy initiation in the late referrals compared with the early referrals^42^. It was previously reported in a study of 32,084 early CKD patients from the NHIRD in Taiwan that an early CKD P4P intervention resulted in a significantly better prognosis in all-cause mortality among matched participants in both P4P programs^43^. Furthermore, the analysis of advanced CKD cases in the present study confirmed that the P4P program for pre-ESRD care education is a crucial tool to improve survival and patient outcomes. Besides, we further analyzed the causes of all-cause mortality in patients with advanced CKD and observed that there were lower events of ischemic heart disease, heart failure, arrhythmia, out-of-hospital cardiac arrest, cerebrovascular accident, infectious disease, and DM complications in those patients receiving pre-ESRD care education. We infer that its main advantages reside in helping patients understand their disease, ameliorating conditions during treatment, and offering strategies to improve patients’ knowledge and outcomes.

### Limitations of this study

This study minimized the selection bias and confounding factors by matching the control group for age, sex, comorbidities, and medications. To correct the confounding bias, we applied a propensity score from the baseline population to match the diversity in characteristics between P4P and non-P4P groups. Despite its strengths and novelty, this study has some limitations that require clarifications. First, the NHIRD does not provide individual laboratory data for further analysis. Second, personal habits and lifestyle cannot be obtained from the NHIRD.

## Conclusions

This study indicated that the P4P program for pre-ESRD care education is an optimal strategy for achieving a higher quality nephrology care, as it was related to reduce the incidence of complications related to hemodialysis, hospitalization rate, and all-cause mortality. These findings suggested that patient education maybe plays an important role in the improvement of pre-ESRD care by reaching patients outside the traditional health care setting. The positive results of the education program are a slower disease progression, lifestyle changes, and the achievement of interdisciplinary care education in group settings.

## Supplementary Information

Supplementary Information.

## Abbreviations

CKD: Chronic kidney disease
DM: Diabetes mellitus
ESRD: End-stage renal disease
NHIRD: National Health Insurance Research Database
P4P: Pay for performance
