# Supplementary material for: Association of pre-ESRD care education with patient outcomes in a 10-year longitudinal study of patients with CKD stages 3–5 in Taiwan
Source: Sci Rep. 2021 Nov 19;11:22602. doi: 10.1038/s41598-021-01860-9 (PMC8604975; doi:10.1038/s41598-021-01860-9)
Supplement: Supplementary file 1 — Supplementary Information. [file 41598_2021_1860_MOESM1_ESM.docx]

**Supplemental Table 1.** The International Classification of Diseases, Ninth Revision, Clinical Modification (ICD-9-CM), National Health Insurance (NHI) Code, and Prescribed Medications Classified Based on Anatomical Therapeutic Chemical (ATC) Classification.

| **Stduy population** | **ICD-9-CM, NHI code, ATC code, and definition** |
| --- | --- |
| CKD, stage Ⅲ (moderate) | 585.3 |
| CKD, stage Ⅳ (severe) | 585.4 |
| CKD, stage Ⅴ, excluding requiring chronic dialysis | 585.5 |
| **Interventions** |  |
| pre-ESRD P4P program | P3402C-P3405C |
| **Outcome** |  |
| ESRD on HD events | Dialysis cost > 0 |
| Hospitalization | Due to CKD |
| Mortality | All-caused |
| Ischemic heart disease | 410-414 |
| Heart failure | 428 |
| Valvular heart disease | 424 |
| Arrhythmia | 427 |
| Out-of-hospital cardiac arrest | 427.5, 798.2 |
| Cerebrovascular accident | 430-438 |
| Infectious disease | 001-139 |
| Cancer | 140-239 |
| Chronic obstructive lung disease | 490-496 |
| Diabetic complication | 250 |
| **Comorbidities** |  |
| Diabetes mellitus | 250.0-250.7 |
| Hypertension | 401-405 |
| Hyperlipidemia | 272 |
| Myocardial infarction | 410, 412 |
| Congestive heart failure | 428 |
| Cerebrovascular disease | 430-437 |
| Chronic pulmonary disease | 490-496, 505, 506.4 |
| Chronic liver disease | 456-456.21, 571.2, 571.4-571.6, 572.2-572.8 |
| Peptic ulcer disease | 531-534 |
| Dementia | 290.0, 290.10-290.13, 290.20-290.21, 290.3. 290.40-290.43, 290.8-290.9, 331.0 |
| Medications | 6 months prior to the index dates |
| ACEI/ARB | C09 |
| Beta-2 blocker | C07 |
| Diuretic | C03 |
| CCB | C08 |
| Antiplatelet drug | B01AC |
| Statin | C10AA |
| NSAID | M01AA-M01AC, M01AE, M01AG-M01AH, M01AX |
| Steroid | H02A-H02C |
| DPP4is | A10BH, A10BD07-A10BD08, A10BD10-A10BD11 |
| Metformin | A10BA |
| Thiazolidinedione | A10BG |
| Sulfonylureas | A10BB |
| Alpha-glucosidase inhibitor | A10BF |
| Insulin | A10A |

Abbreviations: ATC, anatomical therapeutic chemical; ACEI, angiotensin-converting enzyme inhibitor; ARB, angiotensin receptor blocker; CKD, chronic kidney disease; CCB, calcium channel blockers; DDP4is, dipeptidyl peptidase-4 inhibitors; ESRD, end stage renal disease; HD, hemodialysis; ICD-9-CM, international classification of diseases, ninth revision, clinical modification; NHI, national health insurance; NSAID, nonsteroidal anti-inflammatory drug; P4P, pay for performance;
